# Severe Insulin Resistance in a Patient Treated With Nivolumab and Brentuximab-Vedotin for Hodgkin Lymphoma

**DOI:** 10.1210/jcemcr/luad121

**Published:** 2023-11-03

**Authors:** Elif Tama, Meghan Black, Muhamad Alhaj Moustafa, Maria D Hurtado

**Affiliations:** Division of Endocrinology, Diabetes, Metabolism, and Nutrition, Department of Medicine, Mayo Clinic, Jacksonville, FL 32256, USA; Division of Endocrinology, Diabetes, Metabolism, and Nutrition, Department of Medicine, Mayo Clinic, Jacksonville, FL 32256, USA; Division of Hematology and Oncology, Department of Medicine, Mayo Clinic, Jacksonville, FL 32224, USA; Division of Endocrinology, Diabetes, Metabolism, and Nutrition, Department of Medicine, Mayo Clinic, Jacksonville, FL 32256, USA

**Keywords:** insulin resistance, hyperglycemia, immunotherapy, brentuximab-vedotin, nivolumab, cytokine release, hemophagocytic lymphohistiocytosis, Hodgkin lymphoma

## Abstract

This is a case of a 26-year-old male patient, with relapsing Hodgkin lymphoma, treated with nivolumab and brentuximab-vedotin, who was admitted with hyperglycemia and severe insulin resistance requiring approximately 2000 units of intravenous insulin per day. He had no prior diagnosis of diabetes. He was eventually diagnosed with massive cytokine release and hemophagocytic lymphohistiocytosis that led to multi-organ failure and death. The mechanisms behind the hyperglycemia with severe insulin resistance remain unclear but are possibly related to hyperinflammation and immune dysregulation resulting from massive cytokine release. Nivolumab among other immunotherapeutic agents, brentuximab-vedotin, and lymphoid malignancies are rare but known risk factors for massive cytokine release and hemophagocytic lymphohistiocytosis.

## Introduction

Cancer treatments, including glucocorticoids, L-asparaginase, and immune checkpoint inhibitors, are known to cause hyperglycemia via different mechanisms, such as by insulin resistance and decreased insulin production and secretion [1]. The degree of hyperglycemia varies depending on the drug and other risk factors (eg, personal and family history of diabetes and excess adiposity). However, in most cases, hyperglycemia responds to glucose-lowering therapy, may resolve after oncologic treatments are completed or discontinued, and therefore may not require long-term therapy [1]. We present a case of hyperglycemia with severe insulin resistance requiring approximately 2000 units of intravenous (IV) insulin daily in the setting of Hodgkin lymphoma treated with nivolumab, an immune checkpoint inhibitor, and brentuximab-vedotin, an antibody-drug conjugate. In the literature, there are only 3 cases of cancer-related hyperglycemia with severe insulin resistance with daily insulin requirements as high as in this patient, all in patients using brentuximab-vedotin [2-4]. This case contributes to the existing literature of the adverse and severe metabolic derangements that can occur with the use of cancer treatments.

## Case Presentation

Our inpatient endocrinology service was consulted for a case of hyperglycemia with severe insulin resistance and possible diabetes ketoacidosis in a 26-year-old White male patient with relapsed Hodgkin lymphoma 6 years after initial diagnosis. He presented to the emergency department 9 days after receiving the first cycle of brentuximab-vedotin and nivolumab combination. On presentation, he reported chest pain, diffuse myalgia, abdominal and back pain, dry mouth, nausea, vomiting, and poor oral intake for the 2 days prior. At the time of admission, his vital signs were remarkable for tachycardia, hypotension, and tachypnea. His weight was 135 kg and his body mass index (BMI) 41.5 kg/m^2^. The physical examination revealed dry oral mucous membranes and diffuse abdominal and muscle tenderness. The exam was otherwise normal. Initial laboratory evaluation was remarkable for hyperglycemia with a blood glucose of 458 mg/dL/25.4 mmol/L (normal < 140 mg/dL and < 7.8 mmol/L, respectively), mild transaminitis with an aspartate aminotransferase (AST) level of 105 U/L and an alanine aminotransferase (ALT) level of 87 U/L (normal ranges, 8-48 U/L and 7-55 U/L, respectively), hypokalemia of 2.9 mmol/L (normal range, 3.6-5.2 mmol/L), hyponatremia of 132 mmol/L (normal range, 135-145 mmol/L), and hypocalcemia of 6.7 mg/dL/1.67 mmol/L (normal ranges, 8.6-10 mg/dL and 2.2-2.7 mmol/L, respectively) in the setting of hypoalbuminemia of 2.8 mg/dL (normal range, 3.5-5 g/dL). The patient was admitted to the Internal Medicine service unit for further evaluation.

The patient did not have a history of diabetes mellitus. His family history included type 2 diabetes and obesity in his mother, and obesity and hyperlipidemia in his father.

## Diagnostic Assessment

The initial laboratory assessment is summarized in Table 1. As noted, there was significant hyperglycemia. The arterial blood gasometry did not reveal acidosis, specifically metabolic acidosis, therefore ruling out the possibility of diabetes ketoacidosis as a culprit for hyperglycemia. His β-hydroxybutyrate level was slightly elevated at 1.8 mmol/L (normal <0.4 mmol/L), possibly due to poor oral intake during the 2 days preceding his admission. Furthermore, there was no evidence of hyperosmolar hyperglycemic syndrome. As nivolumab is associated with immune-mediated (ie, type 1) diabetes, a C-peptide level and a type 1 diabetes antibody panel were ordered. The C-peptide level was elevated, ruling out insulin deficiency and reflecting increased insulin secretion, an appropriate pancreatic β-cell response to hyperglycemia. His anti-glutamic acid decarboxylase (GAD)-65 antibody was mildly elevated at 0.04 nmol/L (normal level <0.02 nmol/L), which in this case may represent propensity to autoimmune-mediated diabetes later, a side effect of immunotherapeutic agent nivolumab. The rest of type 1 diabetes–associated antibodies including insulin antibodies and islet antigen-2 antibodies were undetectable. His glycated hemoglobin (HbA1c) on admission was elevated at 6.2%, in the range of prediabetes, but nondiagnostic given his anemia and hematologic malignancy. A pituitary hormone evaluation was unremarkable with normal thyrotropin (TSH), free thyroxine (T4), and insulin-like growth factor (IGF) levels. A random afternoon cortisol was elevated at 39 mcg/dL/1076 nmol/L (normal ranges, 2.5-12 mcg/dL and 69-331 nmol/L, respectively), consistent with acute response. This evaluation ruled out hypophysitis and thyroid and adrenal diseases, endocrinopathies also associated with nivolumab use. While sodium was numerically low, after correction for hyperglycemia it was in the normal range. The other electrolyte abnormalities could have been attributed to poor oral intake and vomiting prior to the admission and/or increased sympathetic activity due to acute severe illness upon presentation. Acute cardiac, pulmonary, gastrointestinal, and infectious causes for his presentation were ruled out with a normal electrocardiogram, imaging, and laboratory work up.

**Table 1.** Initial laboratory results

	International system (SI) units (normal range)	Conventional units (normal range)
White blood cell	5.8 (3.4-9.6 ×10^9^/L)	5.8 (3.4-9.6 ×10^9^/L)
Hemoglobin	**6.45** (8.7-11.2 mmol/L)	**10.4** (13.2-16.6 g/dL)
Platelet	**133** (135-317 ×10^9^/L)	**133** (135-317 ×10^9^/L)
Neutrophil	4.55 (1.56-6.45 ×10^9^/L)	4.55 (1.56-6.45 ×10^9^/L)
Glucose	**25.4** (< 7.8 mmol/L)	**458** (< 140 mg/dL)
Calcium	**1.67** (2.2-2.7 mmol/L)	**6.7** (8.6-10 mg/dL)
Sodium, Na^+^	**132** (135-145 mmol/L)	**132** (135-145 mmol/L)
Potassium, K^+^ (mmol/L)	**2.9** (3.6-5.2 mmol/L)	**2.9** (3.6-5.2 mmol/L)
Chloride, Cl^−^ (mmol/L)	**94** (98-107 mmol/L)	**94** (98-107 mmol/L)
Bicarbonate, HCO_3_^−^	**16** (22-29 mmol/L)	**16** (22-29 mmol/L)
Anion gap	14 (7-15 mmol/L)	14 (7-15 mmol/L)
Blood urea nitrogen	**<0.71** (2.1-8.5 mmol/L)	**<2** (8-24 mg/dL)
Creatinine	68.97 (53-106 μmol/L)	0.78 (0.74-1.35 mg/dL)
eGFR	>90 (>=60 mL/min)	>90 (>=60 mL/min/BSA)
Lactate	**6.8** (0.5-2.2 mmol/L)	**61.26** (4.5-19.8 mg/dL)
C-peptide	**7.7** (0.17-0.83 nmol/L)	**23.4** (1.1-4.4 ng/mL)
HbA1c	**44** (20-38 mmol/L)	**6.2** (4.0-5.6%)
Insulin antibodies	0.00 (<0.02 U/mL)	0.00 (<0.02 U/mL)
Islet antigen-2 antibodies	0.00 (<0.02 U/mL)	0.00 (<0.02 U/mL)
Anti-GAD65 antibodies	0.04 (<0.02 U/mL)	0.04 (<0.02 U/mL)
β-hydroxybutyrate	**1.8** (<0.4 mmol/L)	**1.8** (<0.4 mmol/L)
Total cholesterol	2.5 (<5.2 mmol/L)	98 (<200 mg/dL)
Triglycerides	**13.9** (<1.7 mmol/L)	**539** (<150 mg/dL)
ALT	**87** (7-55 U/L)	**87** (7-55 U/L)
AST	**105** (8-48 U/L)	**105** (8-48 U/L)
Albumin	**2.8** (3.5-5 g/L)	**2.8** (3.5-5 g/dL)
Ph, ABG	**7.47** (7.35-7.45)	**7.47** (7.35-7.45)
HCO_3_, ABG	**14.6** (22-26 mmol/L)	**14.6** (22-26 mmol/L)
PCO_2_, ABG	**2.75** (4.7-6 kPa)	**20.6** (35-45 mmHg)
PO_2_, ABG	11.5 (10.5-13.5 kPa)	86.3 (83-108 mmHg)

Abbreviations: ABG, arterial blood gas analysis; ALT, alanine aminotransferase; AST, aspartate aminotransferase; eGFR, estimated glomerular filtration rate; GAD65, glutamic acid decarboxylase 65-kilodalton isoform; HbA1c, glycated hemoglobin A1c.

## Treatment

On admission, he was given 2 liters of IV fluids, IV potassium repletion, and 13 units of subcutaneous aspart insulin. Despite these measures, his blood glucose continued to rise to 487 mg/dL (27.1 mmol/L) and then to 539 mg/dL (29.9 mmol/L) after 1 and 2 hours, respectively. Consequently, during the first day of admission, we started IV regular insulin U100 infusion with an initial bolus of 15 units followed by 0.1 units/kg/hour. During the 3 following days, despite the IV insulin infusion and no oral intake, his hyperglycemia persisted, and insulin requirements increased significantly. Table 2 summarizes how insulin dosing was adjusted based on blood glucose levels and the estimated daily insulin requirements. By the second day of admission, he required more than 1000 units of insulin per day.

**Table 2.** Blood glucose management and insulin dosing during the admission

		Glucose levels mg/dL (mmol/L) normal: < 140 mg/dL, < 7.8 mmol/L	IV insulin type	Dose units/kg/h	SQ insulin type	Dose, units/day	Estimated daily insulin requirements
**Day 1**	**0-12 hour**	458-539 (25.4-30 mmol/L)	Regular U100	Bolus of 15 units and 0.1	Aspart	13	594
	**12-24 hour**	>400 (> 22.2 mmol/L)	Regular U100	0.2	Glargine	80	
**Day 2**	**0-24 hour**	310-350 (17.2-19.4 mmol/L)	Regular U100	0.3	Glargine	160	1132
**Day 3**	**0-12 hour**	250-290 (13.9-16.1 mmol/L)	Regular U100	0.6	Glargine	200	1982
	**12-24 hour**	200-230 (11.1-12.8 mmol/L)	Regular U500	0.5			
**Day 4**	**0-18 hour**	200-230 (11.1-12.8 mmol/L)	Regular U500	0.5	Glargine	150	1689
	**18-24 hour**	100-130 (5.6-7.2 mmol/L)	Regular U500	0.4			
**Day 5**	**0-15 hour**	100-130 (5.6-7.2 mmol/L)	Regular U500	0.2	Glargine	60	678

Abbreviations: IV, Intravenous; SQ, Subcutaneous.

While his hyperglycemia slightly improved on the third day, given the high insulin requirements, we switched him from regular insulin U100 to concentrated regular insulin U500. He was also noted to have worsening hypotension, tachycardia, tachypnea, confusion, and agitation. He was intubated and initiated on continuous renal replacement therapy (CRRT). Concomitantly, laboratory evaluation also revealed hypofibrinogenemia (110 mg/dL, 1.1 g/L; normal range, 200-400 mg/dL, 2-4 g/L) hypertriglyceridemia (1366 mg/dL, 15.44 mmol/L; normal range, <150 mg/dL, < 1.7 mmol/L), hyperferritinemia (10 864 ng/mL, 24.4 nmol/L; normal range, 24-336 ng/mL, 0.05-0.75 nmol/L), high soluble interleukin 2 (>4000 pg/mL; normal range, <960 pg/mL), and bicytopenia (thrombocytopenia and anemia), consistent with a diagnosis of hemophagocytic lymphohistiocytosis (HLH). For this, the HLH treatment protocol was instituted and included high dose glucocorticoids and twice weekly etoposide. At the end of the third day, he received 500 mg of methylprednisolone IV and 200 mg of etoposide IV. Plasmapheresis was initiated concomitantly to remove cytokines and other inflammatory mediators from the circulation.

During the fourth and fifth days of admission, the dose of methylprednisolone was increased to 1000 mg IV daily. In spite of the increasing glucocorticoid doses, glucose levels normalized, and insulin requirements started to decrease gradually. Although hyperglycemia was controlled, his clinical status continued to deteriorate.

## Outcome and Follow-Up

Our patient remained in critical status. He died from multi-organ failure on the fifth day of admission. Notably, in spite of the high doses of glucocorticoids, blood glucose levels normalized on the fourth day of admission. The normalization of blood glucose levels was likely driven by the use of plasmapheresis and the subsequent removal of inflammatory mediators, thereby improving insulin sensitivity.

## Discussion

Our patient represents a case of hyperglycemia with severe insulin resistance in the setting of a lymphoid malignancy treated with nivolumab and brentuximab-vedotin. Nivolumab and brentuximab-vedotin combination is an effective treatment for patients with relapsed or refractory Hodgkin lymphoma. Both agents have been associated with hyperglycemia.

**Figure 1.**
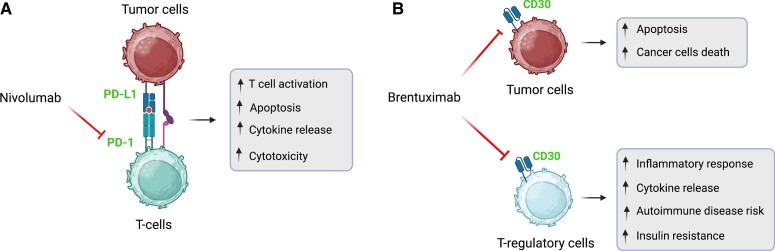
Mechanism and effect of nivolumab (A) and brentuximab (B).

Nivolumab, an immune checkpoint inhibitor, blocks the interaction between programmed cell death protein 1 (PD-1) receptor and its ligand (PD-L1) [5]. PD-1 receptor blockade by nivolumab increases: (i) T-cell response and cytokine secretion, thereby enhancing antitumoral response; and (ii) autoimmune-related inflammation in tissues, thereby increasing the risk of autoimmune disorders (Fig. 1A) [5]. As such, nivolumab use has been associated with autoimmune-mediated destruction of pancreatic β cells, resulting in type 1 diabetes mellitus [5]. Although there was an initial concern for autoimmune-related diabetes ketoacidosis secondary to nivolumab treatment in our patient, laboratory evaluation did not support the diagnosis. Notably, our patient had positive GAD65 antibodies that could have potentially put him at risk of developing autoimmune-mediated diabetes later.

Brentuximab-vedotin is a CD30 directed antibody-drug conjugate. CD30 is expressed on Hodgkin lymphoma cells and T-regulatory cells. As a result, brentuximab-vedotin decreases tumor burden and inhibits T-regulatory cells, thereby increasing cytokine release, enhancing inflammatory response, and inducing insulin resistance (Fig. 1B) [5]. While hyperglycemia has been reported in up to 8% of patients taking brentuximab-vedotin, there are scarce data on the severity and etiology of hyperglycemia, except for 3 case reports of patients presenting with severe hyperglycemia and diabetes ketoacidosis but without type 1 diabetes–associated autoantibodies [2-4].

Given the association between immunotherapy and hyperglycemia, the 2021 American Society of Clinical Oncology guidelines recommend that in all patients using nivolumab and other immune checkpoint inhibitors, providers should monitor glucose levels before starting immunotherapy, before each treatment, and at all follow-up visits for at least 6 months [6]. To date, no guidelines exist for patients using brentuximab-vedotin.

The particularity of this case lies in the severity of hyperglycemia, refractory to intensive insulin therapy, requiring up to 1982 units of insulin per day. To the best of our knowledge, this has not been described with nivolumab treatment. As opposed to nivolumab, brentuximab-vedotin use has been associated with hyperglycemia and severe insulin resistance [2-4]. As previously stated, to date, only 3 cases of brentuximab-vedotin associated with severe hyperglycemia and insulin resistance have been reported [2-4]. All required insulin doses greater than 1000 units per day and all 3 presented with diabetic ketoacidosis [2-4]. One had evidence of massive cytokine release and passed away during the hospital admission from multi-organ failure, like our patient [2]. The 2 surviving patients did not require long-term insulin [2-4].

The etiology of hyperglycemia and high insulin requirements in this case is unclear, but possibly related to massive cytokine release and/or HLH, both present in our patient. Massive cytokine release leads to systemic immune dysregulation and has been rarely reported with brentuximab-vedotin and nivolumab [2, 7, 8]. While massive cytokine release could be attributed to medication toxicity, it could also be secondary to HLH. HLH is an aggressive but rare life-threatening syndrome characterized by cytopenia and progressive multiple-organ failure [9]. HLH results from excessive immune activation and has been reported in association with malignancies, most commonly lymphoid malignancies [9]. However, HLH in patients with malignancy is often triggered by processes that alter immune homeostasis, such as infections, chemotherapy, and immunotherapy [9]. Our patient had 3 risk factors for massive cytokine release, including his lymphoid malignancy, the use of immunotherapy, and the use of an antibody-drug conjugate.

No literature exists on the association between massive cytokine release and severe insulin resistance. We believe that from the pathophysiologic standpoint, these 2 can be linked, as cytokine release and inflammation have been shown to induce insulin resistance in adipose tissue, skeletal muscle, and liver, via inhibition of insulin signal transduction pathways [10]. Perhaps our patient's excess adiposity and possible impaired glucose metabolism contributed to his presentation. To date, there are no reports or evidence in the literature suggesting that excess adiposity or preexistent impaired glucose metabolism (ie, prediabetes or diabetes) are risk factors for severe insulin resistance induced by HLH/cytokine release syndrome. However, among the 3 reported cases of severe hyperglycemia and insulin resistance associated with brentuximab-vedotin use, 1 patient had prediabetes and 2 had BMIs in the obesity category. Further research is needed to explore the underlying links between excess adiposity and impaired glucose metabolism as risk factors for insulin resistance induced by HLH/cytokine release syndrome, as these patients may need to be monitored closely. Finally, while one could have thought that the high glucocorticoid doses that our patient received for HLH may have contributed to insulin resistance, severe insulin resistance with high insulin requirements preceded glucocorticoid initiation.

In summary, we present a rare case of hyperglycemia and severe insulin resistance in a patient with Hodgkin lymphoma treated with brentuximab-vedotin and nivolumab. Despite escalating insulin therapy, his hyperglycemia persisted. The underlying mechanisms for hyperglycemia with severe insulin resistance remain unclear but possibly involve massive cytokine release as a treatment-related toxicity and/or secondary to HLH. Massive cytokine release led to immune system dysregulation and hyperinflammation that eventually caused multi-organ failure. Further research is needed to better understand the connection between immunotherapy, cytokine release syndrome, and insulin resistance. For now, endocrinologists should be aware of this serious metabolic complication in patients undergoing treatment with the combination of brentuximab-vedotin and nivolumab for a lymphoid malignancy.

## Learning Points

- Hyperglycemia with severe insulin resistance can occur as a rare but serious adverse event in patients with lymphoid malignancies treated with brentuximab-vedotin and nivolumab.
- Massive cytokine release could be a culprit for hyperglycemia and severe insulin resistance and could have occurred as a rare treatment-related toxicity and/or secondary to hemophagocytic lymphohistiocytosis.
- The management of hyperglycemia may require aggressive insulin therapy escalation, with daily insulin requirements reaching more than 1000 units.

## Contributors

All authors made individual contributions to authorship. M.D.H., M.B., and M.A.M. participated in the diagnosis and management of the case. M.D.H., E.T., and M.B. participated in the preparation of the manuscript. All authors reviewed and approved the final draft.

## Data Availability

Data sharing is not applicable to this article as no datasets were generated or analyzed during the current study.

## Abbreviations

BMI: body mass index
GAD65: glutamic acid decarboxylase 65-kilodalton isoform
HLH: hemophagocytic lymphohistiocytosis
IV: intravenous
PD-1: programmed cell death protein 1
